# Immunogenicity of an Inactivated COVID-19 Vaccine in People Living with HIV in Guangxi, China: A Prospective Cohort Study

**DOI:** 10.3390/v16091481

**Published:** 2024-09-18

**Authors:** Yuting Wu, Xinwei Wang, Yunxuan Huang, Rongfeng Chen, Yuexiang Xu, Wudi Wei, Fengxiang Qin, Zongxiang Yuan, Jinming Su, Xiu Chen, Jie Liu, Liufang Wen, Minjuan Shi, Tongxue Qin, Yinlu Liao, Beibei Lu, Xing Tao, Cuixiao Wang, Shanshan Chen, Jinmiao Li, William J. Liu, Li Ye, Hao Liang, Junjun Jiang

**Affiliations:** 1 Guangxi Key Laboratory of AIDS Prevention and Treatment, School of Public Health, Guangxi Medical University, Nanning 530021, China; wyt1661989446@163.com (Y.W.); wangxinweivicky@163.com (X.W.); ahbzyzx123@163.com (Z.Y.); sujinming@stu.gxmu.edu.cn (J.S.); shiminjuan@stu.gxmu.edu.cn (M.S.);; 2 Guigang Center for Disease Control and Prevention, Guigang 537100, China; ggaids@126.com (Y.H.);; 3 Joint Laboratory for Emerging Infectious Diseases in China (Guangxi)-ASEAN, Life Sciences Institute, Guangxi Medical University, Nanning 530021, China; 4 National Institute for Viral Disease Control and Prevention, Chinese Center for Disease Control and Prevention, Beijing 102206, China; liujun@ivdc.chinacdc.cn

**Keywords:** PLWH, SARS-CoV-2, inactivated COVID-19 vaccine, immunogenicity

## Abstract

The inactivated COVID-19 vaccine has demonstrated high efficacy in the general population through extensive clinical and real-world studies. However, its effectiveness in immunocompromised individuals, particularly those living with HIV (PLWH), remains limited. In this study, 20 PLWH and 15 HIV-seronegative individuals were recruited to evaluate the immunogenicity of an inactivated COVID-19 vaccine in PLWH through a prospective cohort study. The median age of the 20 PLWH and 15 HIV-seronegative individuals was 42 years and 31 years, respectively. Of the PLWH, nine had been on ART for over five years. The median anti-SARS-CoV-2 S-RBD IgG antibody level on d_224_ was higher than that on d_42_ (8188.7 ng/mL vs. 3200.9 ng/mL, *P* < 0.05). Following COVID-19 infection, the antibody level increased to 29,872.5 ng/mL on d_re+90_, 12.19 times higher than that on d_300_. Compared with HIV-seronegative individuals, the antibody level in PLWH was lower on d_210_ (183.3 ng/mL vs. 509.3 ng/mL, *P* < 0.01), while there was no difference after d_224_. The symptoms of COVID-19 infection in PLWH were comparable to those in HIV-seronegative individuals. In this study, the inactivated COVID-19 vaccine demonstrated good immunogenicity in PLWH. The protective benefit of booster vaccinations for PLWH cannot be ignored. Implementing a booster vaccination policy for PLWH is an effective approach to providing better protection against the COVID-19 pandemic.

## 1. Introduction

SARS-CoV-2 is the pathogen responsible for COVID-19. It primarily targets the respiratory system, resulting in mild infections. In severe cases, it can progress to acute respiratory distress syndrome, leading to multiple organ failure and mortality [1]. As of 12 April 2023, the COVID-19 pandemic has resulted in over 762 million confirmed cases and more than 6.897 million deaths worldwide [2]. Although several clinical trials [3,4,5] and real-world studies [6] have demonstrated the effectiveness of different vaccines against COVID-19 infection, these studies have not provided sufficient information on the immunogenicity of the vaccine in immunocompromised individuals. Some studies have assessed the immune response to COVID-19 vaccination in immunocompromised populations, particularly in individuals who have undergone solid organ transplantation [7], those receiving immunosuppressive therapy [8], individuals with multiple myeloma [9], and those with cancer [10]. However, limited research has evaluated the immunogenicity of inactivated COVID-19 vaccination in HIV-infected individuals, especially considering the significant impact of HIV infection on an individual’s immune status and behavior, as indicated in other diseases [11].

HIV infection is a leading cause of immunodeficiency worldwide, primarily destroying the immune system [12]. By the end of 2021, UNAIDS reported that approximately 38.4 million people were living with HIV [13]. In China, the cumulative number of people living with HIV (PLWH) was 1.223 million by the end of 2022 [14]. Guangxi is located in Southwest China, bordering Southeast Asia. Due to its unique geography and complex population movements, the HIV/AIDS epidemic in Guangxi remains serious, with reported cases ranking among the top three in China [15]. Additionally, PLWH experience higher rates of comorbidities and age-related illnesses [16]. A systematic review and meta-analysis of 22 studies found that PLWH had a 24% higher risk of SARS-CoV-2 infection and a 78% higher risk of mortality from COVID-19 compared to HIV-seronegative individuals [17]. PLWH face the dual challenge of HIV and COVID-19 infection, highlighting the urgent need for effective prevention and protection measures.

Studies have shown that patients with other immunodeficiency diseases, such as cancer [18] and rheumatism [19], can still produce high levels of anti-SARS-CoV-2 S-RBD IgG antibodies after receiving the COVID-19 vaccine. Many mRNA and adenoviral vector COVID-19 vaccines, such as the BNT162b2 COVID-19 mRNA vaccine [20,21,22] and the ChAdOx1 nCOVID-19 vaccine [4,23], were immunogenic and safe in PLWH. However, research on inactivated vaccines against COVID-19 remains relatively limited. It is worth investigating whether inactivated vaccines exhibit good immunogenicity in PLWH. A non-randomized cohort study conducted in Hubei Province provided preliminary evidence regarding the immunogenicity of inactivated vaccines in PLWH [24]. Furthermore, a subsequent cross-sectional study in Beijing supported these findings [25]. However, their study observed PLWH for only a short period after vaccination and did not track them from the time they were unvaccinated until they received all three doses of the vaccine or even after a COVID-19 infection. This limitation restricts the dynamic assessment of immune responses over time in PLWH.

Therefore, we selected a cohort of 20 PLWH undergoing antiretroviral therapy (ART) and 15 HIV-seronegative individuals as the control group for this study. We followed up with study subjects regularly from 20 July 2021 to 20 March 2023 and collected their samples at various time points after vaccination. We analyzed the dynamic changes in the levels of anti-SARS-CoV-2 S-RBD IgG antibodies produced by PLWH after receiving the inactivated COVID-19 vaccine to assess their immune response. At the same time, we evaluated the protective effect of a booster dose of the inactivated COVID-19 vaccine in PLWH by comparing IgG antibody levels between the PLWH group and the control group after administration of the booster dose and recovery from COVID-19 infection. Our study represents a pioneering prospective cohort investigation. This study comprehensively evaluates the immunogenicity of the inactivated COVID-19 vaccine in PLWH from pre-vaccination through post-vaccination completion. It provides a scientific basis for advocating the necessity of COVID-19 vaccination and booster doses in response to the COVID-19 pandemic and promotes the development and optimization of vaccination strategies for PLWH.

## 2. Methods

### 2.1. Ethics Statement

This study was approved by the Ethics and Human Subjects Committee (EHSC) of Guangxi Medical University (No. 20210153).

### 2.2. Study Subjects

With the help of the Guigang Center for Disease Control and Prevention, we recruited a cohort of 20 PLWH who met the predefined inclusion and exclusion criteria. The inclusion criteria were as follows: (1) age ≥ 18 years; (2) confirmed HIV-positive status; (3) absence of COVID-19 infection; and (4) duration of ART treatment ≥ 6 months. The exclusion criteria included: (1) major chronic diseases such as cardiac, renal, pulmonary, endocrine, metabolic, or autoimmune diseases; (2) concurrent infection with hepatitis B/C virus, syphilis, or tuberculosis; (3) hypersensitivity to vaccines and vaccine components; and (4) pregnancy or lactation. Additionally, we recruited 15 HIV-seronegative individuals as controls for this study to assess the effect of HIV infection status on the levels of antibodies produced by comparing the differences in antibody levels between the PLWH and controls. The HIV-seronegative individuals were required to be seronegative for HIV, following inclusion criteria (1) and (3) and the exclusion criteria above. All participants signed the informed consent forms.

### 2.3. Vaccine Information

In this study, subjects were vaccinated with the inactivated COVID-19 vaccine CoronaVac (Vero cells), developed by Beijing Sinovac Zhongwei Biotechnology Co., Ltd., and administered intramuscularly at 0.5 mL per dose. The vaccine was a killed whole virus vaccine, retaining its antigenicity to stimulate an immune response. The batch numbers of the three doses administered to PLWH were the first dose (Lot#: 202106031Z), the second dose (Lot#: 202107094Z), and the booster dose (Lot#: 202112116S). The batch numbers of the vaccine administered to the healthy control group were the first dose (Lot#: 202104010B), the second dose (Lot#: 202105056F), and the booster dose (Lot#: 202111221K).

### 2.4. Information Collection

Information regarding the study subjects, including demographic information such as age, occupation, marital status, the route of infection, ART regimens, and duration of treatment for PLWH, was obtained during the inclusion process, while height and weight were measured. The CD4^+^ T-cell counts of PLWH were obtained by labeling blood cells with anti-CD4-FITC and anti-CD3-PE, collecting the cells via flow cytometry, and gating the analysis to identify CD3+CD4+ lymphocytes, thereby calculating the absolute counts of CD4^+^ T-cells. The COVID-19 infection was detected by collecting nasopharyngeal or oropharyngeal swab samples for nucleic acid testing. A positive result indicated an active COVID-19 infection, while a negative result indicated that the subject was either not infected with COVID-19 or had recovered from it. Symptoms of the COVID-19 infection were collected via face-to-face questionnaires. Fever was defined as an axillary temperature exceeding 37.3 °C. Symptoms such as cough, sore throat, and headache were classified according to medical diagnostic criteria. In terms of infection status, “No symptoms” indicated the absence of any discomfort. “Mild symptoms” referred to conditions that had little impact on daily life and allowed patients to continue their activities, including a slight increase in body temperature (higher than normal but not meeting the criteria for fever), mild malaise or fatigue, and mild headache. “Ordinary symptoms” were more severe than mild symptoms, requiring patients to adjust daily activities but not hospitalization, and included persistent fever, noticeable dry cough, severe muscle or joint pain, headache, and sore throat. “Severe and critical symptoms” required hospitalization, medical intervention, and urgent medical care and included a high fever that did not subside, severe respiratory distress, significant hypoxemia, and respiratory failure.

### 2.5. Sample Collection

**Figure 1:**
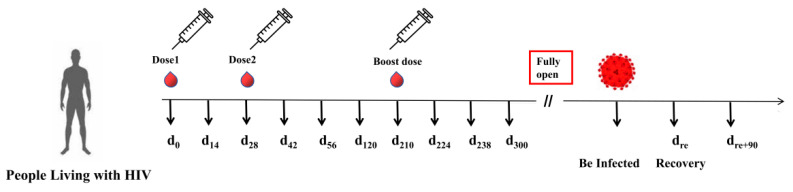
Timeline of peripheral blood sample collection in the cohort of PLWH receiving the inactivated COVID-19 vaccine.

To investigate the dynamics of antibody production and durability in PLWH, we collected peripheral blood samples from participants at various time points. The first dose of the vaccine for PLWH was administered on 20 July 2021 (d_0_); the second dose was on 17 August 2021 (d_28_); and the booster dose was on 16 February 2022 (d_210_). Blood samples from PLWH were collected on the following days: prior to the first dose (d_0_), day 14 (d_14_), day 42 (d_42_), day 56 (d_56_), day 120 (d_120_), day 210 (d_210_), day 224 (d_224_), day 238 (d_238_), day 300 (d_300_), and day 90 after recovery from COVID-19 infection (d_re+90_) (Figure 1). In addition, we followed up with the control group and collected their blood samples on d_210_, d_224_, d_238_, d_300_, and d_re+90_. 

Protocol of the prospective cohort study: PLWH were recruited prior to vaccination and received three doses of the inactivated COVID-19 vaccine. Peripheral blood samples were collected at various time points: d_0_, d_14_, d_42_, d_56_, d_120_, d_210_, d_224_, d_238_, d_300_, and d_re+90_.

### 2.6. Plasma Separation

Peripheral blood samples were collected from study subjects via the antecubital vein using vacuum blood collection tubes containing EDTA at predetermined time points. Samples were promptly processed, and plasma was separated by centrifugation. Subsequently, the upper plasma portion was transferred into sterile 1.5 mL EP tubes, labeled, and stored at −80 °C for future testing.

### 2.7. Anti-SARS-CoV-2 S-RBD IgG Antibody Detection

All samples and reagents were equilibrated at room temperature for 30 min before the experiment. Based on preliminary testing, samples were diluted at various time points after vaccination with ratios ranging from 1:10 to 1:20,000. According to the provided instructions, each sample was tested in duplicate wells using commercial ELISA kits (Proteintech, Wuhan, China), which are commonly used to detect anti-SARS-CoV-2 S-RBD IgG antibodies and have been employed in several studies [26,27,28]. Absorbance values were measured at 450 nm and 630 nm using a microplate reader, and antibody concentration was determined based on these readings. A four-parameter logistic curve fit was used to establish the most appropriate standard curve, with an *R*^2^ ≥ 0.99 considered acceptable.

### 2.8. Statistical Analysis

Descriptive statistics were used to analyze the general characteristics of the study population. As the data did not follow a normal distribution, quantitative variables such as age and BMI were presented as medians and interquartile ranges (IQR), while categorical variables such as gender, ethnicity, and occupation were expressed as frequencies or percentages. The rank-sum test for randomized block design (Friedman’s test), adjusted for significance using the Bonferroni method, was employed to assess differences in IgG antibody concentrations at various time points. The Wilcoxon rank-sum test and Kruskal–Wallis H test were used to compare and analyze differences in different demographic characteristics and antibody levels between PLWH and HIV-seronegative individuals. A logistic regression analysis was conducted to explore the relationship between antibody levels and various demographic characteristics, with specific assignment instructions provided in Table S1. The odds ratio (OR), 95% confidence interval (CI), and *P*-value were used to identify potential influencing factors. Fisher’s exact test was employed to compare differences in categorical variables. Spearman’s rank correlation analysis was conducted to explore the relationship between IgG antibody concentration and the demographic characteristics of PLWH. Statistical analysis was performed using SPSS (version 26.0) software, and figures were created using GraphPad Prism 8. A significance level of *P* < 0.05 was considered statistically significant.

## 3. Results

### 3.1. Subject Characteristics

A total of 20 PLWH and 15 HIV-seronegative controls were included in this study. The PLWH group had a median age of 42 years (IQR: 37, 50), while the control group had a median age of 31 years (IQR: 29, 34). The median body mass index (BMI) for PLWH was 24.24 kg/m^2^ (IQR: 21.39, 27.24), while the BMI for the controls was 20.50 kg/m^2^ (IQR: 18.82, 22.04). There were statistically significant differences (*P* < 0.05) in gender, age, occupation, educational level, and BMI between the two groups. There were no statistically significant differences (*P* < 0.05) in ethnicity or marital status between the two groups (Table 1). For the PLWH group, the median baseline CD4^+^ T-cell count was 431.5 cells/μL (IQR: 381.25, 562.25). Additionally, 80% (16/20) of the patients had a CD4^+^ T-cell count above 350 cells/μL, 85% (17/20) acquired HIV through heterosexual transmission, 75% (15/20) were treated with a combination of NRTIs and NNRTIs, and 45% (9/20) had been undergoing treatment for more than 5 years.

### 3.2. The Conversion Rate of Anti-SARS-CoV-2 S-RBD IgG Antibodies after Vaccination Is Not Associated with Different Characteristics in PLWH

Several factors, including vaccine dosage, gender, age, and immunodeficiency, may influence the overall effectiveness of vaccination. In our study, 17 out of 20 PLWH (85%) demonstrated the production of anti-SARS-CoV-2 IgG antibodies within 14 days after receiving the second dose of the vaccine, classifying them as the IgG-positive group. However, the remaining three individuals (15%), referred to as the IgG-negative group, did not produce anti-SARS-CoV-2 IgG antibodies after receiving the second dose of the vaccine. Nonetheless, all three individuals underwent seroconversion and tested positive for IgG antibodies after receiving the third booster dose. Analysis of clinical characteristics between the IgG-positive and IgG-negative groups revealed no significant differences (*P* > 0.05) in gender, age, ethnicity, occupation, BMI, route of infection, CD4^+^ T-cell count, ART regimen, and duration of ART (Table 2).

### 3.3. The Administration of a Booster Dose or Infection with COVID-19 after Vaccination Can Quickly Boost Antibody Titers in PLWH

**Figure 2:**
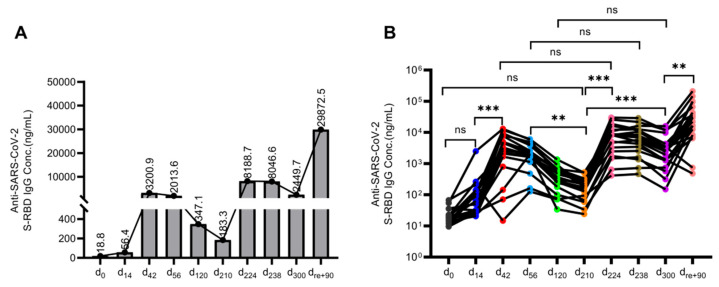
Temporal dynamics of antibody levels in PLWH following inactivated COVID-19 vaccination. (**A**) Changes in median antibody concentration at different time points in PLWH after vaccination and COVID-19 infection. The dots represent median antibody concentrations. The value indicates the specific median antibody concentrations. (**B**) IgG antibody titers of individuals in PLWH are represented by dots. Antibody levels were compared at different time points after vaccination and recovery from the COVID-19 infection using Friedman’s test. The Bonferroni method was used to adjust the test size. *P′* < 0.005 was considered statistically significant. ** *P′* < 0.005, *** *P′* < 0.001, and “ns” indicates no statistical difference.

We observed a modest increase in IgG antibodies targeting the SARS-CoV-2 S-RBD region after the first vaccine dose in PLWH. However, following the second dose, the median IgG antibody concentration peaked at 3200.9 ng/mL on d_42_ and gradually declined over time (Figure 2A). By d_56_, d_120,_ and d_210,_ median antibody concentrations had decreased to 2013.6 ng/mL, 347.1 ng/mL, and 183.3 ng/mL, respectively. There was a statistically significant difference (*P* < 0.005) in antibody levels between d_56_ and d_210_. Remarkably, following the administration of the third booster dose, antibody concentrations on d_224_, d_238_, and d_300_ were 8188.7 ng/mL, 8046.6 ng/mL, and 2449.7 ng/mL, respectively. These values represented a 2.56-fold, 3.99-fold, and 7.06-fold increase compared to the levels observed on d_42_, d_56_, and d_120_, respectively. The antibody concentration showed a substantial 44.67-fold increase on d_224_ and a significant 13.36-fold increase on d_300_ (*P* < 0.001) compared to the levels on d_210_. Following the adjustment of COVID-19 prevention and control measures, the study population experienced successive infections. Notably, the median antibody concentration surged to 29,872.5 ng/mL on d_re+90_, representing a remarkable 12.19-fold increase compared to the level on d_300_ (*P* < 0.005) (Figure 2B).

### 3.4. The Antibody Levels in PLWH after Vaccination or COVID-19 Infection Are Not Influenced by Various Characteristics

**Figure 3:**
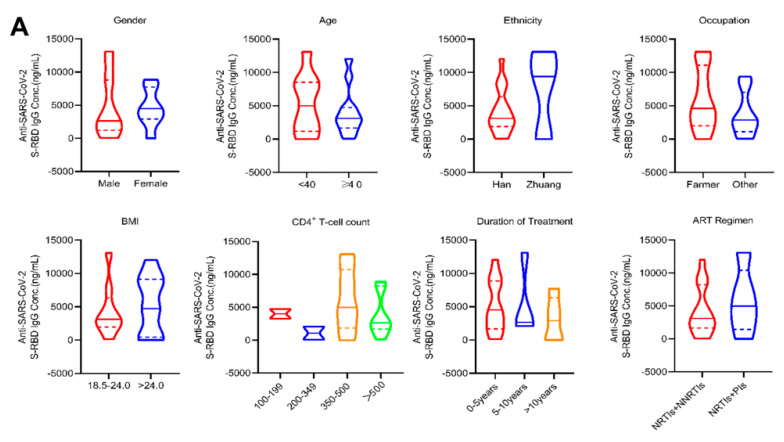

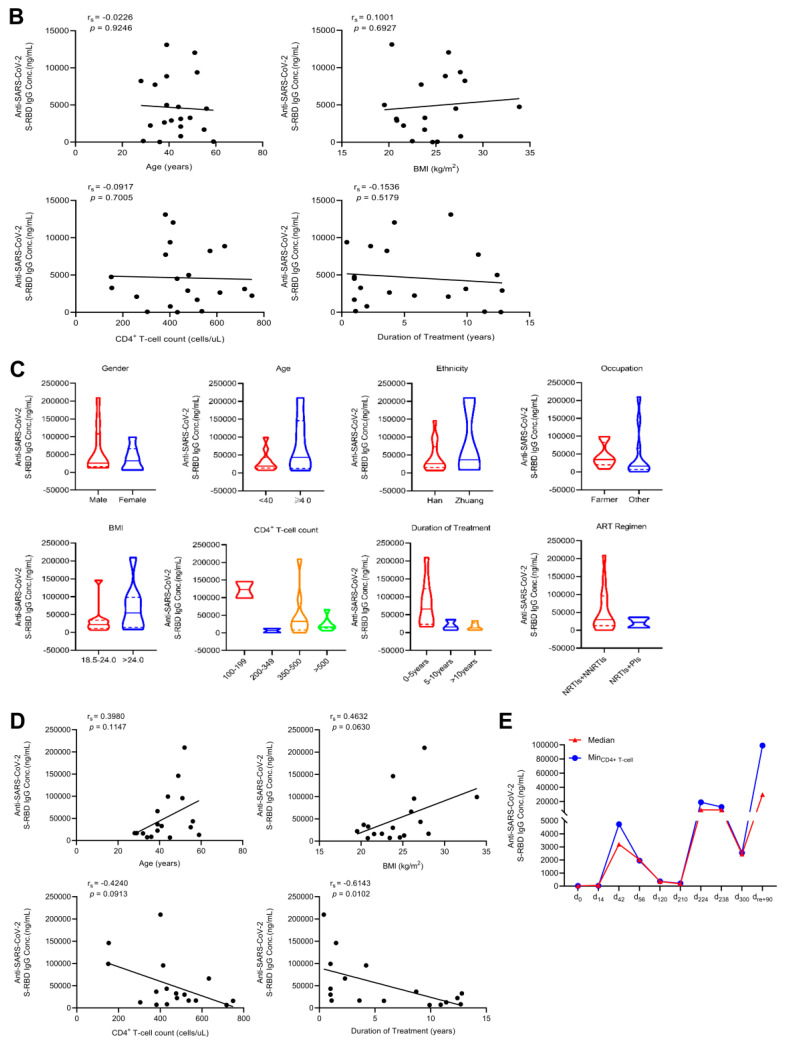
Antibody levels in PLWH with different characteristics after the second dose of vaccination and the COVID-19 infection. (**A**,**C**) The violin plots show the differences in antibody levels by gender, age, ethnicity, occupation, BMI, CD4^+^ T-cell count, duration of treatment, and the ART regimen in PLWH on d_42_ (**A**) and d_re+90_ (**C**); (**B**,**D**) Correlation analysis between age, BMI, CD4^+^ T-cell count, duration of treatment, and antibody levels in PLWH on d_42_ (**B**) and d_re+90_ (**D**). (**E**) The line chart shows changes in antibody levels of the individual with the lowest CD4^+^ T-cell count and in the median antibody levels in PLWH. Comparison of antibody levels with different characteristics was conducted using the Wilcoxon rank-sum test and the Kruskal–Wallis H test. Correlation analysis was performed using Spearman’s rank correlation. Only *P*-values indicating a significant difference were marked.

The analysis of various demographic and clinical characteristics among PLWH in terms of antibody levels after vaccination showed no significant differences in gender, age, ethnicity, occupation, BMI, CD4^+^ T-cell count, duration of treatment, or ART regimen (*P* > 0.05) (Figure 3A). Furthermore, the correlation analysis for age, BMI, CD4^+^ T-cell count, and antibody concentration revealed no significant correlations (*P* > 0.05) (Figure 3B). 

Following COVID-19 infection in PLWH, no significant differences in antibody concentrations were observed based on gender, age, ethnicity, occupation, BMI, CD4^+^ T-cell count, duration of treatment, and the ART regimen (*P* > 0.05) (Figure 3C). Correlation analysis demonstrated a significant negative correlation between the duration of treatment and antibody concentration, as indicated by Spearman’s rank correlation coefficient (r_s_ = −0.6143, *P* = 0.0102). A longer duration of treatment was associated with a more stable level of antibody production in response to SARS-CoV-2 stimulation. The correlation analysis also included age, BMI, and CD4^+^ T-cell count, but no significant correlations were observed (*P* > 0.05) (Figure 3D). Additionally, we compared the post-vaccination antibody levels of the individual with the lowest CD4^+^ T-cell count (151 cells/μL) to the median antibody levels in PLWH. It was found that the individual’s antibody levels approached or even exceeded the median levels (Figure 3E).

### 3.5. PLWH Can Produce Antibody Levels Similar to Those of HIV-Seronegative Individuals after a Booster Vaccine and Following Infection

**Figure 4:**
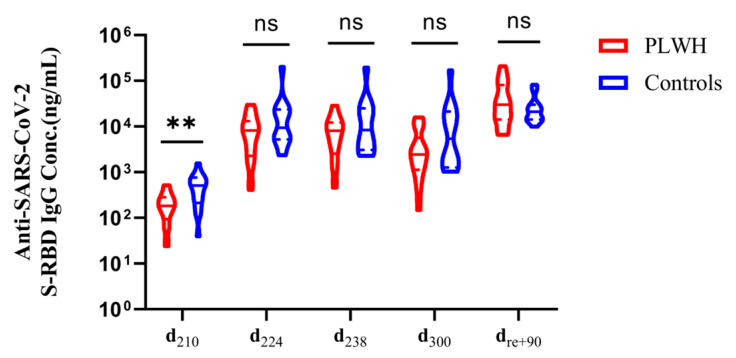
Antibody levels in PLWH and controls following booster vaccination and COVID-19 infection. The violin plots illustrate the comparative analysis of antibody levels in PLWH and controls after administering the booster vaccine and COVID-19 infection, as assessed by the Wilcoxon rank sum test. ** *P* < 0.01, and “ns” indicates no statistical difference.

We compared the antibody levels on d_210_ as well as on d_224_, d_238_, d_300_, and d_re+90_ between PLWH and the controls. After the booster dose, the antibody level in PLWH increased significantly to 8188.7 ng/mL on d_224_, representing a 44.67-fold increase from 183.3 ng/mL on d_210_. Furthermore, the antibody concentration of d_re+90_ was 29,872.5 ng/mL, which was 12.19 times higher than the concentration of d_300_ at 2449.7 ng/mL. In the control group, the average antibody concentration on d_210_ was 509.3 ng/mL. This concentration increased significantly to 9411.1 ng/mL on d_224_, representing an 18.49-fold increase from d_210_. On d_re+90_, the mean antibody concentration was 20,986.3 ng/mL, a 3.90-fold increase compared to the level on d_300_ (5385.7 ng/mL). Notably, the median antibody concentration of PLWH on d_re+90_ was slightly higher than that in the control group. This observation may be attributed to the immune system of PLWH, which enables a rapid and robust antibody response upon encountering viral stimulation. Additionally, the median antibody concentration in the controls on d_210_ was 509.3 ng/mL, 2.78 times higher than that in PLWH, which was 183.3 ng/mL (*P* < 0.01). However, there were no significant differences in antibody levels between PLWH and controls on d_224_, d_238_, d_300,_ or d_re+90_ (Figure 4). 

The results may be influenced by potential confounding factors, such as age and BMI. Consequently, the 35 subjects were divided into two groups based on their total average antibody concentrations: low concentration and high concentration. A logistic regression analysis was used to explore the association between antibody concentrations and various demographic characteristics. The results demonstrated that age, BMI, and other factors had no significant impact on antibody concentration (*P* > 0.05) (Table S2). It was observed that PLWH could achieve protective antibody levels comparable to those of the controls after receiving the booster dose and the COVID-19 infection. In other words, PLWH benefit significantly from the booster dose and COVID-19 infection in terms of antibody production. 

### 3.6. PLWH with COVID-19 Infection Exhibit That Are Comparable to or Milder than Those in HIV-Seronegative Individuals

**Figure 5:**
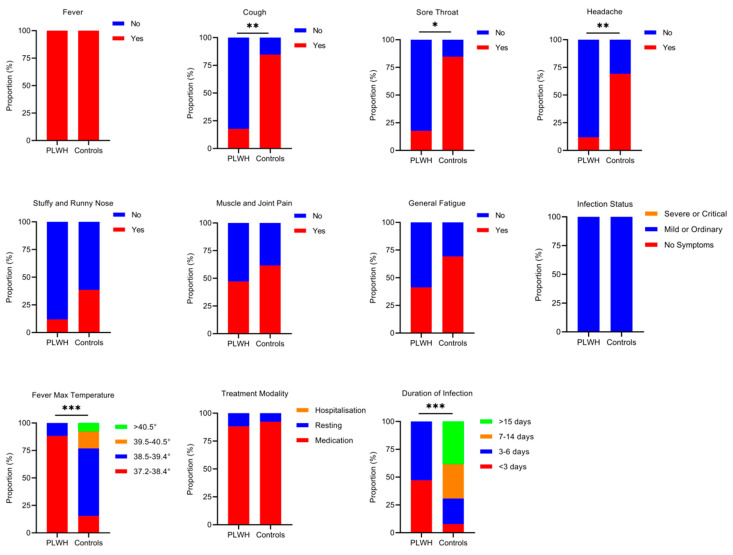
Symptoms of COVID-19 infection in PLWH and the controls. The differences in COVID-19 symptoms between PLWH and controls were analyzed using Fisher’s exact test. The size of the colored squares represents the proportion of various symptoms. Only *P*-values indicating a significant difference are marked. * *P* < 0.05, ** *P* < 0.01; *** *P* < 0.001.

After adjusting the national epidemic prevention and control measures, 17 out of 20 PLWH and 13 out of 15 HIV-seronegative individuals were found to be infected with SARS-CoV-2 in this study. To assess potential differences in symptom severity between PLWH and the controls following COVID-19 infection, we distributed 30-point questionnaires, achieving a 100% response rate with 30 completed questionnaires. The 30 study subjects, including both PLWH and controls, exhibited mild to ordinary symptoms of COVID-19 infection. No fatalities or hospitalization occurred; instead, they were able to recover at home with rest and medication. The symptoms of infection were similar in both groups. There were no significant differences in symptoms, such as fever, stuffy and runny nose, muscle and joint pain, and general fatigue (*P* > 0.05). However, controls exhibited a higher proportion of symptoms, including cough, sore throat, and headache, compared to PLWH. Additionally, controls showed significantly higher values for maximum fever temperature and duration of infection (*P* < 0.05) (Figure 5).

## 4. Discussion

Our study is the first long-term prospective cohort study to track immunogenicity from pre-vaccination through post-COVID-19 recovery. Based on the observed trend in anti-SARS-CoV-2 S-RBD IgG antibody levels in PLWH following inactivated COVID-19 vaccination and infection, our results demonstrated that PLWH exhibited robust immunogenicity in response to the vaccine, with no significant differences in antibody levels observed across various characteristics. Although the level of protective antibodies decreased over time, PLWH who received a booster dose and were subsequently infected with COVID-19 experienced a significant increase in antibody levels. Our study provides new evidence supporting the efficacy of the inactivated COVID-19 vaccine in PLWH.

As of 30 March 2023, there are 199 COVID-19 vaccines in preclinical development and 183 in clinical development worldwide [29]. In China, two inactivated vaccines, the Corona Vac vaccine and the BBIBP-CorV vaccine, have been widely used and show satisfactory immunogenicity in the general population [30,31]. A non-randomized cohort study by Yanmeng Feng [24] found that the HIV-1 viral load among PLWH decreased significantly after BBIBP-CorV vaccination, and their immune responses were comparable to those of healthy individuals. Another single-center study reported serological conversion rates of 94.1%, 99.8%, and 98.6% for PLWH receiving ART after the first, second, and third doses of the BNT162b2 COVID-19 mRNA vaccine, respectively, indicating good immunogenicity [20]. Our study found that the plasma antibody conversion rate in PLWH 14 days after receiving the second dose was 85%, which was lower than the 98% conversion rate reported for healthy individuals receiving the inactivated vaccine during the Phase II clinical trial. The difference may be due to lower immunity and a reduced humoral response to the vaccine in PLWH compared to healthy individuals, or it could result from variations in testing methods. Notably, the antibody conversion rate in PLWH increased to 100% after administration of the booster dose, highlighting the importance of booster vaccination for immunocompromised individuals.

The ideal outcome of vaccination is the development of long-lasting and highly effective protective antibodies, which establish high levels of population immunity and help control the COVID-19 pandemic [32]. However, evidence suggests that PLWH may experience a diminished or shorter duration of vaccine response [33]. Therefore, long-term follow-up studies are needed to assess the persistence of the vaccine response in PLWH. Studies have demonstrated that, 6 months after vaccination, most PLWH still maintain measurable immune responses, which are greater than their pre-vaccination baseline but show evidence of a decline in both humoral and cell-mediated immunity [34]. A booster dose is needed to maintain long-term memory against the SARS-CoV-2 variant. A study published in The Lancet found that, among healthy adults aged 18–59 years, the initial neutralizing antibody response to two doses of CoronaVac declined to near or below the lower limit of seropositivity after 6 months. A third dose of CoronaVac administered 8 months after the second dose resulted in a strong boost in immunity, effectively enhancing the validity, breadth, and likely duration of the memory response to SARS-CoV-2. Compared with the antibody titers 28 days after the second dose, the neutralizing antibody titers had increased approximately three to five times [35]. The detailed immune mechanism underlying the exponential increase in antibody levels following a long-term interval between the second and third doses was determined previously [5]. In our study, similar results were observed in PLWH, where anti-SARS-CoV-2 S-RBD IgG antibodies returned to baseline levels comparable to those observed before vaccination 6 months after the second dose. Antibody concentrations increased significantly 28 days after the booster dose, reaching levels 3.48 times higher than those observed 28 days after the second dose. The booster dose in PLWH not only elevated the conversion rate but also augmented antibody levels.

In addition, our study found that the antibody levels of PLWH 3 months after COVID-19 recovery were significantly higher, showing a 10.86-fold increase compared to those 3 months after the booster dose. Jennifer M. Dan and her team [36] analyzed immune memory to SARS-CoV-2 in 188 COVID-19 cases and found that 88% of subjects were RBD IgG seropositive 6–8 months after out of symptom. Various studies have indicated that patients who recover from COVID-19 may develop longer-lasting protective antibodies and T cells [37]. Research by Goel Rishi R et al. [38] also showed that individuals who had not been infected by SARS-CoV-2 required two doses of vaccine to achieve optimal increases in antibodies. Conversely, those who had recovered from COVID-19 could only need a single dose to achieve peak antibody and memory B cell responses, with antibody levels comparable to those of uninfected individuals who had received two doses of the COVID-19 vaccine. This suggests that natural infection with COVID-19 can induce the production of higher titers of more persistent neutralizing antibodies compared to the inactivated COVID-19 vaccine. 

Studies have shown that PLWH may have an impaired immune status, potentially leading to inadequate vaccine-induced immune responses [39,40]. However, growing evidence suggests that PLWH develop a humoral immune response to the vaccine similar to that of the general population. A randomized, double-blind, controlled trial conducted in South Africa demonstrated that PLWH exhibited good immunogenicity after receiving the COVID-19 vaccine, with RBD-binding IgG and SARS-CoV-2 neutralizing response patterns comparable to those of HIV-negative, SARS-CoV-2 naive individuals following two doses of the vaccine [23]. John Frater [41] also noted that PLWH, who responded well to ART, did not exhibit significant differences in serological and cell-mediated immune responses compared to HIV-negative individuals after receiving two doses of the vaccine. Our study yielded similar results, with no difference in antibody levels between the two groups after receiving a booster dose and recovering from COVID-19 infection. 

Some studies indicated that factors such as age [42], sex [43], and CD4^+^ T-cell counts [25] were related to differences in immune responses to vaccines. Nonetheless, our long-term follow-up study revealed that PLWH aged 18–59 years with varying CD4^+^ T-cell counts demonstrated a good humoral immune response 14 days after the second dose, with no significant difference in antibody levels produced. Moreover, no significant differences were observed in the levels of S-RBD IgG antibodies among PLWH after two doses of vaccine with respect to gender, age, ethnicity, occupation, BMI, ART regimens, and duration of ART (*P* > 0.05). Logistic regression analysis also showed no significant correlation between the various demographic characteristics and antibody levels. Soto-Nava et al. found no associations between SARS-CoV-2 neutralization and HIV-related variables (CD4^+^ T-cell counts, viral load, number of years in viral suppression, ART regimen) in multivariable analysis [44]. These results suggested that various demographic factors had a minimal impact on IgG antibody levels, indicating that PLWH with different immune statuses can achieve equivalent benefits after receiving the inactivated COVID-19 vaccine. However, there was a negative correlation between the antibody level on d_re+90_ and the duration of treatment. This may be because PLWH with a long treatment period were more likely to have a longer duration of HIV infection and exhibit poorer immune function, thereby impacting the antibody levels produced after vaccination. 

Furthermore, we investigated the differences in symptoms during COVID-19 infection between PLWH and HIV-seronegative individuals. Our findings revealed that fever, cough, and fatigue remained prevalent symptoms of COVID-19 infection after vaccination, similar to those observed in the SARS-CoV-2 infection without vaccination [45]. The controls exhibited a higher proportion of symptoms, including cough, sore throat, and headache, compared to PLWH. This difference may be attributed to the relatively late timing of vaccination in PLWH, particularly the shorter interval between the booster vaccination and COVID-19 infection, suggesting a more pronounced protective effect of the vaccine. In contrast, the controls were vaccinated earlier and experienced a greater decline in pre-infection antibodies, leading to an increased incidence of COVID-19 symptoms. Remarkably, none of the PLWH experienced any exacerbations of AIDS due to the COVID-19 infection. After the COVID-19 infection, the symptoms of PLWH were comparable to those of the controls. There were no fatalities or hospitalizations, and recovery was achieved through self-administered medication, home monitoring, and rest. However, a cohort study showed that HIV infection was an independent prognostic factor for poor outcomes of COVID-19 infection, suggesting that PLWH with low CD4^+^ T-cell counts experience a more severe COVID-19 clinical course than those who are HIV-seronegative [46]. It is important to note that some studies have not found a direct association between HIV status and COVID-19 severity but instead highlight the critical role of comorbidities in determining COVID-19 outcomes. A case-control study in Spain found that hospitalized PLWH had a higher risk of death compared to HIV-seronegative individuals, primarily related to the presence of comorbidities rather than virological, immunological, or ART factors [47]. Inciarte’s study also showed that the clinical presentation, severity rate, and mortality of PLWH with COVID-19 infection were not dependent on any HIV-related or ART-related factors [48]. In our study, none of the PLWH reported significant comorbidities and developed protective antibodies after vaccination, which may explain their milder symptoms after COVID-19 infection and also highlight the good immunogenicity and protective effect of the inactivated vaccine in PLWH. This suggests that the inactivated COVID-19 vaccine offers similar protection to both PLWH and HIV-seronegative individuals. Fatima Samar’s study also indicated that non-vaccinated SARS-CoV-2 patients experienced significantly more intensive care hospitalizations and higher mortality than fully vaccinated patients [49], which is consistent with our findings. In other words, even though the antibody titers gradually decline following vaccination, the immune response can be rapidly recalled upon subsequent viral stimulation to prevent disease progression. 

This study has several limitations. Firstly, the sample size was small and did not include PLWH aged 60 years and older. Additionally, we did not collect certain data on PLWH, such as viral loads, which may influence antibody titers. Secondly, the use of questionnaires could not eliminate recall bias, as symptom information was gathered 3 months after the subjects had recovered from COVID-19. Thirdly, the HIV-seronegative individuals did not match with the PLWH group in terms of demographic information, leading to discrepancies in several variables.

In conclusion, the inactivated COVID-19 vaccine demonstrates good immunogenicity in PLWH. The immune responses produced by PLWH after vaccination and COVID-19 infection are comparable to those in healthy individuals. Although the antibody-positive conversion rate is slightly lower and the persistence of protective antibodies in PLWH is poor, they can be recalled with a booster dose of the vaccine. Therefore, implementing a booster dose vaccination strategy in PLWH is crucial to enhancing the protection of the immunocompromised population against worsening AIDS progression.

## Figures and Tables

**Table 1:** Demographic characteristics at baseline in the PLWH and control groups.

	PLWH (*n* = 20)	Controls (*n* = 15)	*P* ^#^
Gender			0.041
Male	13 (65.0)	4 (26.7)	
Female	7 (35.0)	11 (73.3)	
Age (years)			0.046
<40	9 (45.0)	12 (80.0)	
40~59	11 (55.0)	3 (20.0)	
Ethnicity			0.129
Han	17 (85.0)	9 (60.0)	
Zhuang	3 (15.0)	6 (40.0)	
Occupation			<0.001
Farmer	8 (40.0)	0 (0.0)	
Worker	4 (20.0)	0 (0.0)	
Others	8 (40.0)	15 (100.0)	
Marital Status			0.598
Married	12 (60.0)	8 (53.3)	
Single	7 (35.0)	7 (46.7)	
Divorce or Widowhood	1 (5.0)	0 (0.0)	
Educational Level			<0.001
Senior Secondary and above	2 (10.0)	15 (100.0)	
Junior Secondary	11 (55.0)	0 (0.0)	
Primary school and below	7 (35.0)	0 (0.0)	
BMI			0.038
18.5~24.0	9 (45.0)	13 (86.7)	
>24.0	9 (45.0)	2 (13.3)	
Unknown	2 (10.0)	0 (0.0)	
Route of Infection			-
Heterosexual trans	17 (85.0)	/	
Homosexual trans	1 (5.0)	/	
Unknown	2 (10.0)	/	
CD4^+^ T-cell count (cells/µL)			-
≥500	7 (35.0)	/	
350–499	9 (45.0)	/	
200–349	2 (10.0)	/	
100–199	2 (10.0)	/	
ART Regimen			-
NRTIs + NNRTIs	15 (75.0)	/	
NRTIs + PIs	5 (25.0)	/	
Duration of Treatment (years)			-
<5	11 (55.0)	/	
5~10	4 (20.0)	/	
>10	5 (25.0)	/	

The data were represented by n (%). *P*^#^-values were calculated by a two-tailed Fisher’s exact test.

**Table 2:** Comparison of various characteristics between the IgG-positive and IgG-negative groups of PLWH after the second dose of the vaccine.

	IgG-Positive (*n* = 17)	IgG-Negative (*n* = 3)	*P* ^#^
Gender			1.000
Male	11 (64.7)	2 (66.7)	
Female	6 (35.3)	1 (33.3)	
Age (years)			0.566
<40	7 (41.2)	2 (66.7)	
40–59	10 (58.8)	1 (33.3)	
Ethnicity			0.404
Han	15 (88.2)	2 (66.7)	
Zhuang	2 (11.8)	1 (33.3)	
Occupation			0.838
Farmer	7 (41.2)	1 (33.3)	
Worker	3 (17.6)	1 (33.3)	
Other	7 (41.2)	1 (33.4)	
BMI			1.000
18.5–24.0	8 (53.3)	1 (33.3)	
>24.0	7 (46.7)	2 (66.7)	
Infection Route			0.101
Heterosexual trans	15 (88.2)	2 (66.7)	
Homosexual trans	0 (0.0)	1 (33.3)	
Unknown	2 (11.8)	0 (0.0)	
CD4^+^ T-cell count (cells/μL)			0.579
≥500	6 (35.3)	1 (33.3)	
350–499	8 (47.0)	1 (33.3)	
200–349	1 (5.9)	1 (33.3)	
100–199	2 (11.8)	0 (0.0)	
ART Regimen			1.000
NRTIs+NNRTIs	13 (76.5)	2 (66.7)	
NRTIs+PIs	4 (23.5)	1 (33.3)	
Duration of Treatment (years)			0.176
<5	10 (58.8)	1 (33.3)	
5~10	4 (23.5)	0 (0.0)	
>10	3 (17.6)	2 (66.7)	

The data were represented by n (%), and *P*^#^-values were calculated using the two-tailed Fisher’s exact test.

## Data Availability

All data from this study are included in this manuscript and are available from the primary contract (Junjun Jiang, Email: jiangjunjun@gxmu.edu.cn) upon request.

## Supplementary Materials

The following supporting information can be downloaded at: https://www.mdpi.com/article/10.3390/v16091481/s1, Table S1: Assignment description of logistic regression analysis; Table S2: A multivariate logistic regression model of antibody concentration and demographic characteristics.
