# Supplementary material for: Immunogenicity of an Inactivated COVID-19 Vaccine in People Living with HIV in Guangxi, China: A Prospective Cohort Study
Source: Viruses. 2024 Sep 18;16(9):1481. doi: 10.3390/v16091481 (PMC11437430; doi:10.3390/v16091481)
Supplement: Supplementary file 1 [file viruses-16-01481-s001.zip › viruses-3069415-supplementary.pdf]

Supplementary Table

Table S1

Assignment Description of Logistic Regression Analysis

Factors	Assignment description
Gender	Male=0, Female=1
Age	< 40=0， 40~59=2
Ethnicity	Han=0, Zhuang=1
Occupation	Farmer=0, Worker=1, Others=2
Marital Status	Married=0, Single=1, Divorce or Widowhood=2
Educational Level	Senior Secondary and above=0, Junior Secondary=1, Primary School and below=2
BMI	18.5~24.0=0, > 24.0=1, Unknown=2
HIV Infection	No=0, Yes=1

Table S2

A multivariate logistic regression model of antibody concentration and demographic characteristics

Variables	OR	95% CI for OR		*P*
		Lower	Upper	
Gender				
Male	Reference			
Female	1.353	0.209	8.768	0.751
Age				
<40	Reference			
40~59	0.488	0.026	9.058	0.630
Ethnicity				
Han	Reference			
Zhuang	3.104	0.202	47.771	0.417
Occupation				
Farmer	Reference			
Worker	1.989	0.026	152.88	0.756
Others	1.178	0.011	124.38	0.945
Marital Status				
Married	Reference			
Single	0.575	0.000	——	1.000
Divorce or Widowhood	0.075	0.000	——	1.000
Educational Level				
Senior Secondary and	Reference			
Junior Secondary	0.000	0.000	——	0.999
Primary school and	1.365	0.026	72.332	0.878
BMI				
18.5~24.0	Reference			
>24.0	0.000	0.000	——	1.000
Unknown	0.000	0.000	——	1.000
HIV Infection				
No	Reference			
Yes	0.000	0.000	——	0.999
